# Phylogeographic Assessment Reveals Geographic Sources of HIV-1 Dissemination Among Men Who Have Sex With Men in Kenya

**DOI:** 10.3389/fmicb.2022.843330

**Published:** 2022-03-09

**Authors:** George M. Nduva, Frederick Otieno, Joshua Kimani, Lyle R. McKinnon, Francois Cholette, Paul Sandstrom, Susan M. Graham, Matt A. Price, Adrian D. Smith, Robert C. Bailey, Amin S. Hassan, Joakim Esbjörnsson, Eduard J. Sanders

**Affiliations:** ^1^Department of Translational Medicine, Lund University, Lund, Sweden; ^2^Kenya Medical Research Institute-Wellcome Trust Research Programme, Kilifi, Kenya; ^3^Nyanza Reproductive Health Society, Kisumu, Kenya; ^4^Department of Medical Microbiology, University of Nairobi, Nairobi, Kenya; ^5^Department of Medical Microbiology and Infectious Diseases, University of Manitoba, Winnipeg, MB, Canada; ^6^Centre for the AIDS Programme of Research in South Africa (CAPRISA), Durban, South Africa; ^7^National Microbiology Laboratory at the JC Wilt Infectious Diseases Research Centre, Public Health Agency of Canada, Winnipeg, MB, Canada; ^8^Department of Epidemiology, University of Washington, Seattle, WA, United States; ^9^IAVI, San Francisco, CA, United States; ^10^Department of Epidemiology and Biostatistics, University of California, San Francisco, San Francisco, CA, United States; ^11^Nuffield Department of Medicine, The University of Oxford, Oxford, United Kingdom; ^12^Division of Epidemiology and Biostatistics, University of Illinois Chicago, Chicago, IL, United States

**Keywords:** HIV-1, molecular epidemiology, phylogeographic, MSM, Kenya

## Abstract

HIV-1 transmission dynamics involving men who have sex with men (MSM) in Africa are not well understood. We investigated the rates of HIV-1 transmission between MSM across three regions in Kenya: Coast, Nairobi, and Nyanza. We analyzed 372 HIV-1 partial *pol* sequences sampled during 2006–2019 from MSM in Coast (*N* = 178, 47.9%), Nairobi (*N* = 137, 36.8%), and Nyanza (*N* = 57, 15.3%) provinces in Kenya. Maximum-likelihood (ML) phylogenetics and Bayesian inference were used to determine HIV-1 clusters, evolutionary dynamics, and virus migration rates between geographic regions. HIV-1 sub-subtype A1 (72.0%) was most common followed by subtype D (11.0%), unique recombinant forms (8.9%), subtype C (5.9%), CRF 21A2D (0.8%), subtype G (0.8%), CRF 16A2D (0.3%), and subtype B (0.3%). Forty-six clusters (size range 2–20 sequences) were found—half (50.0%) of which had evidence of extensive HIV-1 mixing among different provinces. Data revealed an exponential increase in infections among MSM during the early-to-mid 2000s and stable or decreasing transmission dynamics in recent years (2017–2019). Phylogeographic inference showed significant (Bayes factor, BF > 3) HIV-1 dissemination from Coast to Nairobi and Nyanza provinces, and from Nairobi to Nyanza province. Strengthening HIV-1 prevention programs to MSM in geographic locations with higher HIV-1 prevalence among MSM (such as Coast and Nairobi) may reduce HIV-1 incidence among MSM in Kenya.

## Introduction

In sub-Saharan Africa, the HIV-1 epidemic among men who have sex with men (MSM) has only recently received attention—and the role of MSM in HIV-1 transmission has been acknowledged (Beyrer et al., 2010; Sanders et al., 2015a; Nduva et al., 2021). In Kenya, the national HIV-1 prevalence is 4.9% in the adult population, but is threefold higher in MSM than in heterosexual men (Kenya National Aids Control Council, 2019; National AIDS and STI Control Programme [NASCOP], 2020). HIV-1 prevalence among MSM in Kenya varies between regions—and ranges from 17.8% in Kisumu (Western Kenya) (Kunzweiler et al., 2017) to 24.5% in Coastal Kenya (Sanders et al., 2007), and from 25.0 to 26.4% in Nairobi (Smith et al., 2021a,b). There is evidence of high mobility of MSM sex workers between regions, which could link HIV-1 transmissions in different regions (Geibel et al., 2008). The Ministry of Health in Kenya through the National AIDS Control Council (NACC) has made efforts to strengthen HIV healthcare services for MSM (Gruskin and Tarantola, 2008; van der Elst et al., 2020). Yet, stigma against male-same-sex practices and policies criminalizing consensual same-sex sexual practices have obstructed progress (Cohen et al., 2013; van der Elst et al., 2013, 2020). In the past, geographic mobility has been shown to play an important role in HIV-1 dispersal (Faria et al., 2014; Grabowski et al., 2020). Taken together, it is possible that spatial differences in the HIV-1 distribution in Kenya combined with geographically mobile MSM sex workers could impact HIV-1 spread among MSM throughout the country (Faria et al., 2014; Grabowski et al., 2020). However, clear data on HIV-1 transmission dynamics within and between MSM in different geographic regions are lacking in Kenya.

HIV-1 transmission dynamics can be assessed by linking sociodemographic, clinical, and behavioral data with HIV-1 sequence data through phylogenetics (Brenner et al., 2007; Volz et al., 2013; Bruhn et al., 2014; Frentz et al., 2014; Pybus et al., 2015; Esbjörnsson et al., 2016; Poon et al., 2016; Ratmann et al., 2016; Hassan et al., 2017; Sallam et al., 2017). While limited HIV-1 sequences have been obtained from blood plasma from MSM living with HIV in Kenya, phylogenetic determination of patterns of HIV-1 transmission among Kenyan MSM suggests extensive MSM HIV-1 clustering (and infrequent HIV-1 mixing between MSM and presumed heterosexuals in the general population) (Bezemer et al., 2014; Hassan et al., 2018; Nduva et al., 2020, 2021, in press). In addition, a phylogenetic study in 2013 reported frequent HIV-1 gene flow between MSM in Coastal Kenya and Nairobi—albeit with small sample size and limited geographic coverage (Bezemer et al., 2014). In the period 2005–2019, more MSM HIV-1 sequences have become available from diverse geographical locations in Kenya, allowing in-depth characterization of evolutionary dynamics in the MSM HIV-1 epidemic in Kenya. Here, we used HIV-1 *pol* data to phylodynamically infer HIV-1 transmission rates among MSM in three different geographic regions in Kenya.

## Materials and Methods

### Study Population

New sequences were generated from blood plasma obtained through studies conducted through the MSM Health Research Consortium—a multisite collaboration between researchers affiliated with KEMRI-Wellcome Trust (KWTRP) in Coastal Kenya, Nyanza Reproductive Health Society (NRHS) in Nyanza, and Sex Workers Outreach Program (SWOP) clinics in Nairobi. These included samples from Coast derived from participants in a prospective observational cohort (2006–2019) (Sanders et al., 2013), samples from Nairobi from a respondent-driven sample survey (Transform, 2017; Smith et al., 2021b), and samples from Nyanza derived from the Anza Mapema cohort (2015–2017) (Kunzweiler et al., 2018).

### HIV-1 *Pol* Sequence Dataset

The HIV-1 *pol* sequences were comprised of 1,020 nucleotides, HXB2 [K03455] positions 2267–3287. HIV-1 RNA was purified from patient blood plasma using the RNeasy Lipid Tissue Mini Kit (QIAGEN) as previously described (Esbjörnsson et al., 2010). Reverse transcription and amplification of partial *pol* gene were performed using the One-Step Superscript III RT/Platinum Taq High Fidelity Enzyme Mix (Thermo Fisher Scientific™) with the *pol*-specific primer pair JA269 and JA272 (Hedskog et al., 2010). First-round PCR products were amplified in a nested PCR with DreamTaq Green DNA Polymerase (Thermo Fisher Scientific™) using *pol-*specific primers JA271 and JA270 (Hedskog et al., 2010). PCR products were sequenced in both directions with the nested PCR primers using the BigDye terminator kit v1.1 (Applied Biosystems), and the sequences were determined on an ABI PRISM 3130xl Genetic Analyzer (Applied Biosystems).

Additional Kenyan HIV-1 *pol* sequences (referred to as published sequences, 2006–2019) were retrieved (October 11th 2021) from the Los Alamos HIV-1 sequence database (Los Alamos National Laboratory, 2019). The combined new and published sequences (referred to as the Kenyan dataset) were annotated with information on sampling dates and geographical area of residence during sampling (i.e., province; Coast, Nairobi, and Nyanza).

### HIV-1 Subtyping

The Kenyan dataset was aligned with the HIV-1 Group M (subtypes A-K + Recombinants) subtype reference dataset (available at the Los Alamos HIV database)^1^ using the MAFFT algorithm in Geneious Prime 2019 (Larkin et al., 2007; Los Alamos National Laboratory, 2019). The resulting alignment was used to construct a maximum-likelihood phylogenetic tree in PhyML using the general time-reversible substitution model with a gamma-distributed rate variation and proportion of invariant sites (GTR+Γ4+I) (Guindon et al., 2005). Branch support was assessed using the Shimodaira–Hasegawa like approximate likelihood ratio test (aLRT-SH) in PhyML, with aLRT-SH ≥ 0.90 considered as significant (Anisimova and Gascuel, 2006; Hassan et al., 2017). Subtypes were assigned based on the Subtype/CRF-resolved phylogeny visualized using FigTree v1.4.4^2^. Subtype assignment was further verified using the REGA HIV-1 Subtyping Tool (v.3.0), and unique recombinant forms (URFs) were detected using the jumping profile hidden Markov model (jpHMM) (Schultz et al., 2009; Pineda-Peña et al., 2013).

### HIV-1 Cluster Analysis

Sequences were grouped into subtype-specific datasets, and a search for related sequences was done for each subtype-specific (A1, C, and D) dataset using the NCBI GenBank BLAST tool, limiting results to the 10 most similar hits per sequence, and retaining the oldest sequence per individual (Kouyos et al., 2010; Esbjörnsson et al., 2016; Sallam et al., 2017). Kenyan sequences and reference sequences were combined and aligned using the MAFFT algorithm in Geneious Prime 2019 (Larkin et al., 2007). Subtype-specific alignments were edited to exclude codon positions associated with drug resistance, and maximum-likelihood phylogenies were reconstructed in PhyML. For each subtype, monophyletic clades with aLRT-SH support ≥0.9 and which were dominated (≥80%) by Kenyan sequences (compared to reference sequences) were defined as Kenyan HIV-1 clusters (Hassan et al., 2017). Clusters were classified based on the number of sequences per cluster into dyads (2 sequences), networks (3–14 sequences), and large clusters (>14 sequences) (Esbjörnsson et al., 2016; Nduva et al., 2020; Abidi et al., 2021).

### Bayesian Phylodynamic and Discrete Phylogeographic Inference

To date clusters and to estimate the effective population size through time (*N*_*e.T*_), Bayesian phylodynamic inference was performed in BEAST 1.10.4 using the Bayesian Skygrid model, an uncorrelated lognormal relaxed clock, and the general time-reversible substitution model with a gamma-distributed rate variation and proportion of invariant sites (GTR+Γ4+I) (Drummond et al., 2005; Baele et al., 2012; Gill et al., 2013; Suchard et al., 2018). Only sequences classified as pure A1, C, and D subtypes were analyzed. BEAST runs were computed with a chain length of 100–300 million generations for each dataset, sampling every 10,000th–30,000th iteration, and discarding the first 10% as burn-in. Convergence was determined in Tracer v.1.7.0 and defined as effective sample sizes (ESS) ≥100 (Suchard et al., 2018). Maximum clade credibility (MCC) trees were summarized using Tree-Annotator v1.8.2 (BEAST suite).

To infer the direction of virus movements between geographic locations from HIV-1 sequence data, a discrete phylogeographic inference was computed using specific locations as independent discrete states (Lemey et al., 2009; Edwards et al., 2011; Faria et al., 2014). Several sensitivity analyses were performed to test the robustness of our data. Firstly, the Kenyan dataset was grouped by subtype (A1, C, and D), and the phylogeographic inference was performed using all the sequences per subtype. Secondly, to reduce sampling bias arising from the unproportionable allocation of sequences per location, sequences in the subtype A1-specific dataset (the largest of the three subtypes) were randomized and subsampled into a dataset with an equal number of sequences per province using in-house Perl scripts (available upon request). Lastly, subtype A1 sequences from Coast province were subsampled uniformly and used to estimate virus migration between three geographically distinct regions in Coastal Kenya (i.e., Mombasa, South Coast, and North Coast).

In the phylogeographic inference, the asymmetric model was adopted (over the alternative symmetric model) as it relaxes the assumption of constant diffusion rates through time to realistically model the location-exchange processes (Edwards et al., 2011; Faria et al., 2014). In addition to estimating the direction of HIV-1 migration, the proportions of forward and reverse rates of migrations between geographic locations were quantified using a robust counting approach (Markov jumps) implemented in BEAST (Minin and Suchard, 2008). Maximum clade credibility (MCC) trees annotated with demographic and epidemiological data were summarized in Tree-Annotator v1.10.4 (BEAST suite) and visualized in FigTree (v1.4.4). Well-supported virus movements and Bayes factors (BFs) assessing statistical support were summarized using SPREAD v1.0.7, and BF ≥ 3 was considered significant (Lemey et al., 2009).

### Statistical Analysis

Continuous data were presented using medians and interquartile ranges (IQRs). Frequencies and percentages were used to describe categorical data. A multivariable logistic regression model was used to assess associations between individual sequence characteristics [e.g., subtype, location of sampling, year (range) of sampling, and source of sequence data—i.e., published or newly generated] and phylogenetic clustering. Statistics and summary plots were done using Stata 15 (StataCorp LLC, College Station, TX, United States) and RStudio (version 1.2.5001) with the packages *yarrr* and *ggplot2* (Wickham, 2016; Phillips, 2017).

### Ethical Consideration

Plasma samples used to generate the new sequences were obtained from ongoing or concluded studies that were also approved by Kenya Medical Research Institute (KEMRI) Scientific and Ethics Review Unit (SERU 3747, 3280, and 3520, and SSC 894). Since published sequences were obtained from an open-access public domain, informed consent was not retrospectively obtained. Instead, we sought approval through a study protocol that was reviewed by KEMRI/SERU (SERU 3547).

## Results

### Study Population, Sequence Dataset, and Subtype Distribution

**FIGURE 1:**
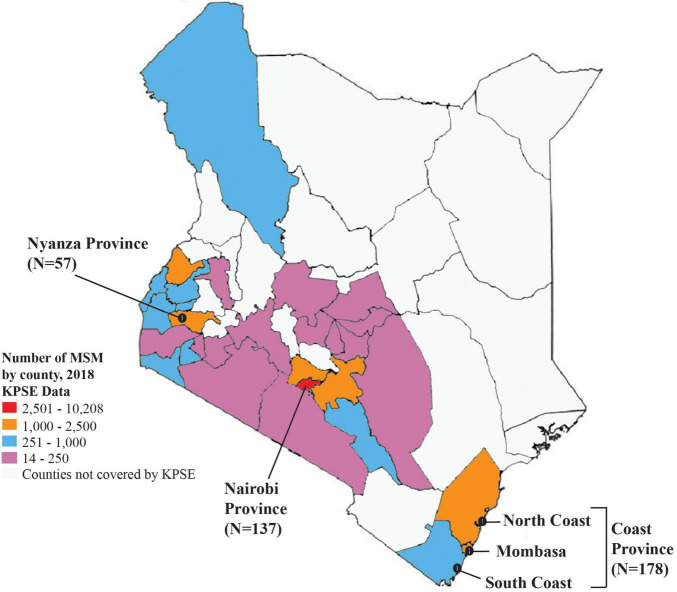
Map of Kenya showing the distribution of sequences in this study. A map of Kenya showing the number of HIV-1 sequences from MSM analyzed in this study, and distribution by different geographic regions. The map is colored based on the estimated number of MSM as mapped at the county level during the 2018 key population size estimates national survey (National AIDS and STI Control Programme [NASCOP], 2019).

**FIGURE 2:**
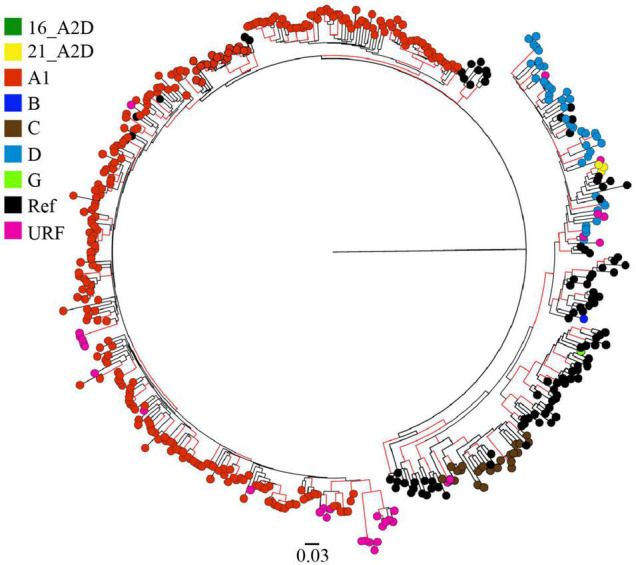
HIV-1 genotypes among 372 MSM sequences from Kenya. Maximum-likelihood phylogenetic tree of 372 HIV-1 *pol* sequences from MSM living with HIV-1 in Kenya (and 194 HIV-1 Group M subtype reference sequences from the Los Alamos HIV database). Branch tips colors correspond to the respective HIV-1 subtype, sub-subtype, or recombinant form as shown in the legend. Branches with aLRT-SH support of more than ≥0.9 are colored red. The tree is drawn to scale, with branch lengths measured in the number of substitutions per site.

Among the 372 HIV-1 partial *pol* sequences analyzed, 213 (57.3%) were generated in this study, and 159 (42.7%) were previously published. The majority (*N* = 178, 47.9%) of the sequences were from Coast province, 137 (36.8%) from Nairobi province, and 57 (15.3%) from Nyanza province (Figure 1, Table 1, Supplementary Figure 1, and Supplementary Tables 1, 2). Sequences belonged to sub-subtype A1 (*N* = 268, 72.0%), subtype D (*N* = 41, 11.0%), subtype C (*N* = 22, 5.9%), subtype G (*N* = 3, 0.8%), CRF 21A2D (*N* = 3, 0.8%), CRF 16A2D (*N* = 1, 0.3%), and subtype B (*N* = 1, 0.3%). Unique recombinant forms (URFs) identified included A1D (*N* = 19, 5.1%), A1C (*N* = 7, 1.9%), D01AE (*N* = 5, 1.3%), A1B (*N* = 1, 0.3%), and DB (*N* = 1, 0.3%, Figure 2).

**TABLE 1:** Distribution of newly generated and published HIV-1 *pol* sequences (*N* = 372) from Kenyan MSM, overall, and by geographic location.

Category	Number of sequences (*N*, %)			
**Geographic region**	**Coast**	**Nairobi**	**Nyanza**	**Total**
**Year (range)**				
2006–2010	117 (65.7%)	1 (0.7%)	0 (0.0%)	118 (31.7%)
2011–2015	32 (18.0%)	1 (0.7%)	19 (33.3%)	52 (14.0%)
2016–2019	29 (16.3%)	135 (98.5%)	38 (66.7%)	202 (54.3%)
**Sequences**				
New	21 (11.8%)	135 (98.5%)	57 (100%)	213 (57.3%)
Published	157 (88.2%)	2 (1.5%)	0 (0.0%)	159 (42.7%)
**Subtype**				
A1	121 (68%)	102 (74.5%)	45 (79%)	268 (72%)
D	22 (12.4%)	13 (9.5%)	6 (10.5%)	41 (11%)
URF	16 (9%)	14 (10.2%)	3 (5.3%)	33 (8.9%)
C	14 (7.9%)	5 (3.7%)	3 (5.3%)	22 (5.9%)
21A2D	0 (0%)	3 (2.2%)	0 (0%)	3 (0.8%)
G	3 (1.7%)	0 (0%)	0 (0%)	3 (0.8%)
16A2D	1 (0.6%)	0 (0%)	0 (0%)	1 (0.3%)
B	1 (0.6%)	0 (0%)	0 (0%)	1 (0.3%)
Total	178 (47.9%)	137 (36.8%)	57 (15.3%)	372 (100%)

*MSM, men who have sex with men; URF, unique recombinant form; CRF, circulating recombinant form.*

### Men Who Have Sex With Men HIV-1 Clusters

Clusters were determined from maximum-likelihood (ML) phylogenies reconstructed for the most prevalent HIV-1 subtypes in the population [subtypes A (A1), C, and D—cumulatively comprising 89.0% of the sequences in the Kenyan dataset]. Non-Kenyan HIV-1 reference sequences were obtained from GenBank based on similarity (where of 931 participant-unique sub-subtype A1 sequences remained after removal of redundancies; 488 for subtype C; and 350 for subtype D). Of 331 (A1, C, and D) sequences in the cluster analysis, 229 sequences (61.2%) formed 46 statistically supported clusters (size range: 2–20 sequences). Dyad/pairs were most common (*N* = 25, 54.4% of all clusters), followed by networks having 3–14 sequences (*N* = 18, 39.1%), and large clusters having more than 14 sequences (*N* = 3, 6.5%). The majority (*N* = 34, 73.9%) were sub-subtype A1 clusters, followed by subtype D (*N* = 8, 17.4%) and subtype C (*N* = 4, 8.7%, Table 2 and Supplementary Figure 2).

**TABLE 2:** The number of Kenyan MSM HIV-1 clusters by cluster size and geographic region.

	Dyads (2 sequences)	Networks (3–14)	Large clusters (≥14)	Total clusters
**Subtype**				
A1	12 (66.7%)	19 (76.0%)	3 (100%)	34 (73.9%)
C	2 (11.1%)	2 (8.0%)	0 (0.0%)	4 (8.7%)
D	4 (22.2%)	4 (16.0%)	0 (0.0%)	8 (17.4%)
**Geographic region**				
Coast	6 (24.0%)	8 (44.4%)	0 (0.0%)	14 (30.4%)
Coast/Nairobi	11 (44.0%)	2 (11.1%)	0 (0.0%)	13 (28.3%)
Nairobi	2 (8.0%)	4 (22.2%)	0 (0.0%)	6 (13.0%)
Nyanza/Nairobi/Coast	2 (8.0%)	0 (0.0%)	3 (100%)	5 (10.9%)
Nyanza	0 (0.0%)	3 (16.67%)	0 (0.0%)	3 (6.5%)
Nyanza/Nairobi	3 (12.0%)	0 (0.0%)	0 (0.0%)	3 (6.5%)
Nyanza/Coast	1 (4.0%)	1 (5.56%)	0 (0.0%)	2 (4.4%)
Total	25 (54.4%)	18 (39.1%)	3 (6.5%)	46 (100%)

*MSM, men who have sex with men. Clusters were classified based on the number of sequences per cluster into dyads (2 sequences), networks (3–14 sequences), and large clusters (>14 sequences).*

### Geographic Stratification of Clustering Patterns

Stratification of clusters by geographic regions showed two distinct clustering patterns. First, some clusters (*N* = 23, 50.0%) had sequences belonging exclusively to one specific province including Coast (*N* = 14, 30.4%), Nairobi (*N* = 6, 13.0%), and Nyanza (*N* = 3, 6.5%) province-exclusive clusters. The remaining clusters (*N* = 23, 50.0%) were mixed between different provinces where HIV-1 mixing between Coast and Nairobi was most common (*N* = 13, 28.3% clusters), followed by mixing between Nyanza, Nairobi, and Coast (*N* = 5, 10.9%), Nyanza and Nairobi (*N* = 3, 6.5%), and Nyanza and Coast (*N* = 2, 4.4%, Table 2 and Supplementary Figure 2). Sequences from Nairobi province were more likely to cluster compared to sequences from Coast province [adjusted odds ratio (aOR) 3.5, 95% confidence interval (CI) 1.2–10.4, *P* = 0.022, Table 3].

**TABLE 3:** Factors associated with HIV-1 clustering among MSM with HIV-1 in Kenya.

Characteristics		Multivariate analysis
		*aOR, (95% CI), *p*-value
Year (range)	2006–2010	Reference
	2011–2015	1.0 (0.4–2.2), 0.937
	2016–2020	1.1 (0.3–3.4), 0.932
Subtype	A1	Reference
	C	0.6 (0.2–1.5), 0.258
	D	1.0 (0.5–2.0), 0.884
Province	Coast	Reference
	Nairobi	3.5 (1.2–10.4), 0.022
	Nyanza	1.8 (0.5–5.9), 0.34
Sequence	Published	Reference
	Newly generated	2.5 (1.7–4.0), <0.001

MSM, men who have sex with men; *aOR, adjusted odds ratio.

### Estimating Effective Population Size Through Time and Dating Clusters

**FIGURE 3:**
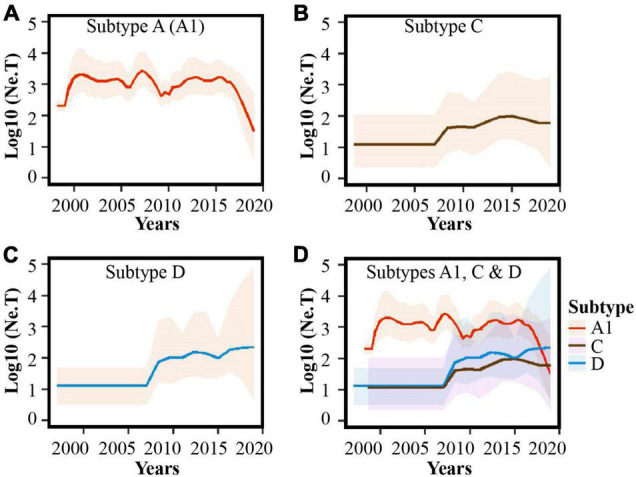
Population dynamics of HIV-1 sub-subtype A1, subtype D, and subtype C lineages among MSM in Kenya. Bayesian Skygrid plots showing population dynamics of the **(A)** HIV-1 sub-subtype A1, **(B)** HIV-1 subtype C, **(C)** HIV-1 subtype D lineages, and **(D)** combined plots for HIV-1 A1, C and D lineages in Kenyan MSM. Median estimates of the number of MSM contributing to new infections are shown as a continuous line in each plot (colored red for sub-subtype A1, brown for subtype C, and blue for subtype D). The shaded area represents the 95% higher posterior density intervals of the inferred effective population size for each lineage.

In-depth phylodynamic analysis indicated that the number of MSM contributing to new HIV-1 A1 infections over time increased exponentially during the early 2000s, followed by a period with some fluctuation (but largely steady) between 2000 and 2017, and mostly decreasing dynamics during recent years (2017–2019, Figure 3A). Likewise, for both subtype C and D lineages, the effective population size increased exponentially during 2007–2008 and has stabilized in recent years (2016–2019; Figures 3B–D).

**FIGURE 4:**
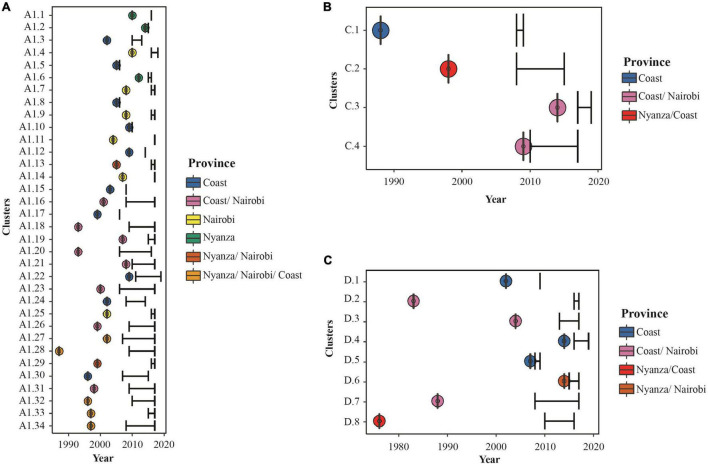
Characteristics and posterior distribution of time to most recent common ancestors estimated for all Kenya clusters. Bayesian tMRCA estimates for **(A)** HIV-1 sub-subtype A1, **(B)** HIV-1 subtype C, and **(C)** HIV-1 subtype D lineages in Kenyan MSM HIV-1 clusters. Dots represent the estimated tMRCA and are colored as per the provinces represented by sequences in each cluster as shown in the legend. Black error bars represent sampling time (with lower interval representing the oldest sampling time per cluster and upper interval representing the most recent sampling time per cluster).

Estimating dates of origins of all clusters indicated that the majority (65%) of transmissions within clusters took place between 2000 and 2014. The oldest sub-subtype A1 cluster had nine MSM from Nyanza, Nairobi, and Coast and had originated during 1987, while the youngest cluster was dated to 2014 among MSM in Nyanza (Figure 4A, Supplementary Figure 3, and Supplementary Table 3). The largest A1 cluster (*N* = 20, 2008–2017) had remained active over 20 years since the estimated time to the most recent common ancestor (tMRCA) in 1997 and was geographically spread out to Nyanza, Nairobi, and Coast provinces. The second-largest A1 cluster (*N* = 19, 2008–2017) originated in 1996 and had sequences from Nyanza, Nairobi, and Coast provinces. The four subtype C clusters originated during 1988, 1998, 2009, and 2014, respectively, while the earliest subtype D cluster originated during 1976 and the youngest during 2014 (Figures 4B,C and Supplementary Table 3). Overall, there was evidence of onward HIV-1 transmission among MSM, within longstanding and geographically diverse HIV-1 networks.

### HIV-1 Migration Between Provinces in Kenya

Ancestral locations and rates in historical virus jumps were first estimated based on all subtype-specific sequences in the Kenyan dataset (i.e., 268 sub-subtype A1, 41 subtype D, and 22 subtype C sequences). Phylogeographic analysis indicated significant support (Bayes Factor, BF ≥ 3) for virus migration from Coast to Nairobi (BF = 3716; subtype A1, BF = 268; subtype C; and BF = 16; subtype D) and from Nairobi to Nyanza (BF = 3716; subtype A1, BF = 43; subtype D, Supplementary Table 4). Exploring temporal trends in virus transitions between geographic provinces summarized from trait-annotated maximum clade credibility trees indicated that the proportion of virus export from Coast to Nairobi increased from 4.2% before 2000 to 14.2% during 2001–2010 and declined to 4.9% during 2011–2020. Likewise, virus export from Nairobi to Nyanza increased from 2.4% in 2000–2010 to 10.8% in 2011–2020, while reverse transitions were rare and occurred only from Nyanza to Nairobi (Supplementary Figures 4, 5 and Supplementary Table 5).

**FIGURE 5:**
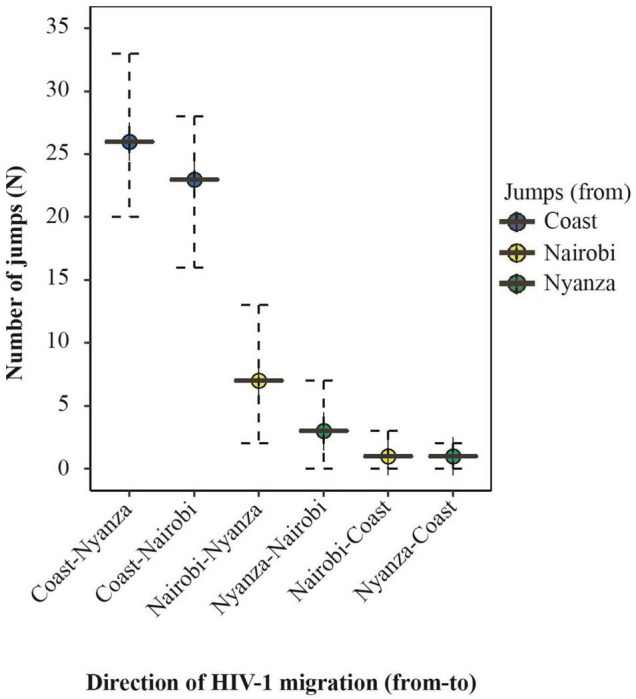
Summary of the expected number of HIV-1 migration between geographic regions in Kenya. Summary of the median number (and 95% HPD interval) of Markov jumps inferred with a uniform sampling of geographic regions. Plots represent HIV-1 exchange between provinces. Plots are colored by the “source” location as shown in the legend. Only statistically significant transitions [Bayes Factor (BF) ≥ 3] are plotted.

A sensitivity analysis with uniform sampling per province was performed to confirm the robustness of the initial phylogeographic inference. The uniformly subsampled dataset comprised 135 HIV-1 sub-subtype A1 sequences (45 sequences each from Nairobi, Mombasa, and Nyanza province). Based on this analysis, there was significant support for HIV-1 migration from Coast to Nairobi (BF = 7766), Nairobi to Nyanza (BF = 1293), and Coast to Nyanza (BF = 336, Table 4). Furthermore, Markov jumps estimates with uniform sampling indicated that the majority (80.3%) of HIV-1 jumps between provinces occurred from Coast to other provinces including jumps from Coast to Nyanza (*N* = 26, 42.6% of all virus jumps between provinces) and from Coast to Nairobi (*N* = 23, 37.7%, Figure 5 and Table 5). There was also some (*N* = 10, 16.4%) virus exchange between Nairobi and Nyanza, such that virus jump Nairobi to Nyanza (*N* = 7, 11.5%) was twofold higher than from Nyanza to Nairobi (*N* = 3, 4.9%, Table 5).

**TABLE 4:** HIV-1 migration rates (Bayes factor, BF ≥ 3) between geographic locations in Kenya.

The direction of migration events (from, to)	Bayes factor (BF)	Posterior probability
**Migration between provinces**		
Coast-to-Nairobi	7766	1
Nairobi-to-Nyanza	1293	1
Coast-to-Nyanza	336	1
Nyanza-to-Nairobi	3	0.7
Nyanza-to-Coast	3	0.7

**TABLE 5:** The number of expected (Markov) jumps inferred for HIV-1 A1 migration between geographic locations.

The direction of migration events (from, to)	Number of HIV-1 jumps (*N*, %)
**Between provinces**	**61 (100%)**
Coast–Nyanza	26 (42.6%)
Coast–Nairobi	23 (37.7%)
Nairobi–Nyanza	7 (11.5%)
Nyanza–Nairobi	3 (4.9%)
Nairobi–Coast	1 (1.6%)
Nyanza–Coast	1 (1.6%)

## Discussion

We found high rates of HIV-1 geographic mixing and a high proportion of HIV-1 sequences exported from the Coast and Nairobi to Nyanza province—implying that the Coast and Nairobi provinces could be major geographic sources of HIV-1 transmission among Kenyan MSM. Of all provinces in Kenya, the Coast and Nairobi provinces have the highest prevalence of HIV-1 among MSM (Kenya National Aids Control Council, 2009). In addition, MSM in Coastal Kenya are known to be highly mobile, and some engage in sex work in different locations across the country (Geibel et al., 2008). Taken together, our findings suggest that regions with the highest HIV-1 prevalence among MSM (such as Coast and Nairobi) may also have disseminated HIV-1 disproportionately to regions with lower HIV-1 prevalence among MSM (such as Nyanza province) in Kenya. s

There are a few presumed mechanisms by which Coastal Kenya may serve as an important source of infections among MSM. One plausible explanation might be that as a very well recognized destination for domestic tourism and sex tourism, MSM (or non-disclosing HET) visit the area for sex tourism, effectively disseminating the virus upon returning from Coast. A second potential determinant could be connected to geographically mobile MSM sex workers—hypothetically, HIV-1 may first be acquired and/or amplified in the Coast, and then exported to other provinces. Thus, the regional difference observed could potentially reflect amplification behavior within Coastal Kenya—and onward spread to other provinces linked to an MSM migration gradient. Data on migration were not available during the current analysis, but future studies may investigate this in detail. Future studies may also potentially investigate potential underlying demographic transitions—speculatively, young MSM sex workers may be drawn to Coast province while older or socially privileged MSM or MSM sex workers may leave the region for other provinces. Overall, implementing HIV-1 prevention and care directed to MSM in Kenya (and considering areas with higher rates of HIV-1 dissemination such as Coast and Nairobi) might reduce ongoing HIV-1 transmission at a countrywide scale, as has been shown in other settings (Bailey et al., 2007; Anderson et al., 2014; Gerberry et al., 2014; McGillen et al., 2016).

The majority (61.2%) of sequences analyzed in this study formed phylogenetically linked HIV-1 clusters, consistent with multiple introductions and ongoing infections among MSM within close networks in Kenya (Skar et al., 2011; Esbjörnsson et al., 2016; Sallam et al., 2017). Half of the clusters comprised sequences collected from MSM from different geographic regions—indicating geographically extensive HIV-1 linkages. High rates of clustering involving HIV-1 in MSM have been reported both in our setting and other higher-income settings and could be linked to an increased risk of infection among MSM within close networks, involving geographically mobile individuals (Geibel et al., 2008; Bezemer et al., 2014; Esbjörnsson et al., 2016; Sallam et al., 2017; Hassan et al., 2018). We estimated that a high proportion (65%) of HIV-1 transmissions occurred between 2000 and 2014 and that several clusters extended over multiple years, suggesting onward HIV-1 transmission among MSM within geographically diverse HIV-1 networks. HIV-1 sequences in this study were not closely related to reference sequences from the global epidemic, implying that the HIV-1 epidemic among MSM in Kenya is sustained locally.

In a broader context, several phylogenetic studies have revealed that the HIV-1 epidemic in Kenya is compartmentalized—where the majority of HIV-1 transmission occurs within risk groups (Bezemer et al., 2014; Nduva et al., 2020, in press). Our recent work at a countrywide scale has demonstrated a minor (8%) proportion of HIV-1 MSM and heterosexual clustering (Nduva et al., in press). Taken together, these studies indicate that ongoing transmission among MSM rarely impacts the general heterosexual HIV-1 epidemic in Kenya. MSM in Kenya have a high burden of HIV risk—to reduce overall HIV-1 incidence in Kenya, there is a need to implement directed HIV-1 prevention and treatment to MSM in Kenya.

The phylodynamic analysis investigating the evolutionary dynamics of the HIV-1 MSM sub-epidemic revealed an exponential increase in the number of infections during the early-to-mid 2000s (for HIV-1 A1, C, and D lineages)—indicative of multiple HIV-1 outbreaks among Kenyan MSM (Skar et al., 2011; Esbjörnsson et al., 2016; Sallam et al., 2017). Interestingly, the effective population size did not decrease following the nationwide introduction and scale-up of combination antiretroviral therapy (ART) in 2004. One potential reason for this is suboptimal access to HIV-1 treatment and prevention services by MSM in Kenya due to fear of legal and social stigma and discrimination (Micheni et al., 2015; Kenya National Aids Control Council, 2019; Stannah et al., 2019). Nevertheless, the effective population size for the dominant strain (HIV-1 A1) showed fewer new infections in recent years (2017–2019)—possibly reflecting earlier ART initiation due to changes in treatment recommendations (National AIDS and STDS Control Programme Ministry of Health, 2016) as well as some impact of risk reduction counseling, adherence support interventions (Möller et al., 2015; Graham et al., 2020), early recognition of acute HIV-1 infections, especially on the Kenyan Coast (Sanders et al., 2014, 2015b; Mugo et al., 2016), and some uptake of pre-exposure prophylaxis targeting MSM in recent years (Graham et al., 2015; van der Elst et al., 2015; Wahome et al., 2018; Kimani et al., 2019). Overall, increasing access to treatment, as well as destigmatization and diversification of providers, may further reduce HIV-1 incidence among MSM (Smith et al., 2021b).

The major strength of our study is the use of HIV-1 sequences from well-characterized acute and early infected MSM cohorts sampled over 14 years in a sub-Saharan African setting. A limitation is that the study had a small sample size, which limited the identification of HIV-1 links in the entire MSM HIV-1 epidemic in Kenya. Incomplete sampling likely resulted in missing links and reduced clustering of HIV-1 sequences (Novitsky et al., 2014). However, our sensitivity analyses before and after controlling for sampling bias indicated more jumps from Coastal Kenya to other provinces (and from Nairobi to Nyanza) than vice versa, indicating the robustness of the analyzed HIV-1 sequence dataset. Another limitation is skewed spatiotemporal sampling and variations in sampling methods between studies, which may have resulted in overrepresentation of some types of location-specific and/or subtype-specific clusters. Indeed, the HIV-1 C and HIV D lineages did not have a decreasing trend in recent years (2017–2019, compared to HIV-1 A1)—the reason for this could be related to skewed sampling over time in various geographic locations in this study. In addition, although the conflation of MSM and transgender people may have relevance for the distinction between sexual network types, we did not have data on gender identity—thus, some transgender people may have been conflated for MSM.

## Conclusion

We demonstrated extensive HIV-1 mixing among MSM in different regions in Kenya, where Coast and Nairobi provinces appear to have been a major source of virus dissemination. We hypothesize that MSM in these provinces may have disseminated HIV-1 disproportionately to MSM in other regions in the country. Increasing PrEP uptake and access to ART among MSM (and destigmatization and diversification of providers) is necessary to reduce ongoing HIV-1 transmission among MSM in Kenya.

## Data Availability Statement

The data presented in the study are deposited in GenBank, accession numbers OM109723-OM109725, OM109756-OM109766, OM109772-OM109799, OM109814-OM109862, OM109879-OM109949, OM110011-OM110019, OM110126-OM110127, OM110136-OM110149, OM110169-OM110170, OM110171, OM110174, OM110178-OM110181, OM110193-OM110194, OM110212-OM110218, OM110229-OM110240, OM110245-OM110246, and OM110272-OM110282.

## Ethics Statement

The studies involving human participants were reviewed and approved by the plasma samples used to generate the new sequences were obtained from ongoing or concluded studies that were also approved by the Kenya Medical Research Institute (KEMRI) Scientific and Ethics Review Unit (SERU 3747, 3280, and 3520, and SSC 894). Since published sequences were obtained from an open-access public domain, informed consent was not retrospectively obtained. Instead, we sought approval through a study protocol that was reviewed by KEMRI/SERU (SERU 3547). The patients/participants provided their written informed consent to participate in this study.

## Author Contributions

GN, AH, ES, and JE conceptualized and designed the study, and provided funding for the study. FO, JK, LM, PS, SG, MP, AS, RB, and ES provided samples from which new sequences used in the study were generated. GN performed lab work and inferential analyses, produced all figures and tables, and wrote the manuscript. FC helped with virus sequencing. All authors reviewed, edited, and approved the manuscript for submission.

## Author Disclaimer

The contents of this manuscript are the responsibility of the authors and do not necessarily reflect the views of USAID or the United States Government, AAS, NEPAD Agency, Wellcome Trust, IAVI, Swedish Research Council, or the United Kingdom Government.

## Conflict of Interest

The authors declare that the research was conducted in the absence of any commercial or financial relationships that could be construed as a potential conflict of interest.

## Publisher’s Note

All claims expressed in this article are solely those of the authors and do not necessarily represent those of their affiliated organizations, or those of the publisher, the editors and the reviewers. Any product that may be evaluated in this article, or claim that may be made by its manufacturer, is not guaranteed or endorsed by the publisher.

## Footnotes

1
http://www.hiv.lanl.gov

2
https://github.com/rambaut/figtree/releases
