# Supplementary material for: Phylogeographic Assessment Reveals Geographic Sources of HIV-1 Dissemination Among Men Who Have Sex With Men in Kenya
Source: Front Microbiol. 2022 Mar 9;13:843330. doi: 10.3389/fmicb.2022.843330 (PMC8959701; doi:10.3389/fmicb.2022.843330)

**SUPPLEMENTARY DATA**

**Files in this Data Supplement:**

Table S1. Distribution of HIV-1 subtypes by year (range) of sampling.

Table S2. Distribution of HIV-1 subtypes by year (range) and geographic province of sampling.

Table S3. Characteristics and posterior distribution of time to most recent common ancestors estimated for all Kenya clusters.

Table S4. Phylogeographic inference of HIV-1 migration rates (Bayes factor, BF≥3) between geographic locations in the full Kenyan dataset.

**Legends for supplementary figures.**

Figure S1. The frequency of HIV-1 subtypes per province and by year (range) of sampling.

Figure S2. The maximum-likelihood tree used to identify HIV-1 clusters.

Figure S3. The maximum clade credibility trees used to date clusters.

Figure S4. The maximum clade credibility tree summary of the Bayesian inference.

# Figure S5. The proportion and dates of HIV-1 transitions between geographic provinces and risk groups.

# **TABLES**

**Table S1. Overall distribution of HIV-1 subtypes by year (range) of sampling.**

| **HIV-1 Subtype** | **Years (Range)** | | | |
| --- | --- | --- | --- | --- |
|  | **2006-2010** | **2011-2015** | **2016-2019** | **Total** |
| **A1** | 84 (31.3%) | 38 (14.2%) | 146 (54.5%) | 268 (72.0%) |
| **D** | 13 (31.7%) | 8 (19.5%) | 20 (48.8%) | 41 (11.0%) |
| **URF** | 9 (27.3%) | 2 (6.1%) | 22 (66.7%) | 33 (8.9%) |
| **C** | 7 (31.8%) | 4 (18.2%) | 11 (50.0%) | 22 (5.9%) |
| **21_A2D** | 0 (0.0%) | 0 (0.0%) | 3 (100.0%) | 3 (0.8%) |
| **G** | 3 (100%) | 0 (0.0%) | 0 (0.0%) | 3 (0.8%) |
| **16_A2D** | 1 (100%) | 0 (0.0%) | 0 (0.0%) | 1 (0.3%) |
| **B** | 1 (100%) | 0 (0.0%) | 0 (0.0%) | 1 (0.3%) |
| **Total** | 118 (31.7%) | 52 (14.0%) | 202 (54.3%) | 372 (100.0%) |

**Table S2. Distribution of HIV-1 subtypes by year (range) of sampling and geographic area of sampling.**

| **Province** | **Subtype** | **Year range (N, %)** | | | **Total (N, %)** |
| --- | --- | --- | --- | --- | --- |
|  |  | **2006-2010** | **2011-2015** | **2016-2019** |  |
| **Coast** | A1 | 84 (71.8%) | 26 (81.3%) | 11 (37.9%) | 121 (100.0%) |
|  | D | 13 (11.1%) | 4 (12.5%) | 5 (17.2%) | 22 (100.0%) |
|  | URF | 8 (6.8%) | 1 (3.1%) | 7 (24.1%) | 16 (100.0%) |
|  | C | 7 (6%) | 1 (3.1%) | 6 (20.7%) | 14 (100.0%) |
|  | G | 3 (2.6%) | 0 (0.0%) | 0 (0.0%) | 3 (100.0%) |
|  | 16A2D | 1 (0.9%) | 0 (0.0%) | 0 (0.0%) | 1 (100.0%) |
|  | B | 1 (0.9%) | 0 (0.0%) | 0 (0.0%) | 1 (100.0%) |
| **Sub-total** |  | 117 (100.0%) | 32 (100.0%) | 29 (100.0%) | 178 (100.0%) |
| **Nairobi** | A1 | 0 (0.0%) | 1 (100%) | 101 (74.8%) | 102 (100.0%) |
|  | URF | 1 (100%) | 0 (0.0%) | 13 (9.6%) | 14 (100.0%) |
|  | D | 0 (0.0%) | 0 (0.0%) | 13 (9.6%) | 13 (100.0%) |
|  | C | 0 (0.0%) | 0 (0.0%) | 5 (3.7%) | 5 (100.0%) |
|  | 21A2D | 0 (0.0%) | 0 (0.0%) | 3 (2.2%) | 3 (100.0%) |
| **Sub-total** |  | 1 (100%) | 1 (100%) | 135 (100.0%) | 137 (100.0%) |
| **Nyanza** | A1 | 0 (0.0%) | 11 (57.9%) | 34 (89.5%) | 45 (100.0%) |
|  | D | 0 (0.0%) | 4 (21.1%) | 2 (5.3%) | 6 (100.0%) |
|  | C | 0 (0.0%) | 3 (15.8%) | 0 (0.0%) | 3 (100.0%) |
|  | URF | 0 (0.0%) | 1 (5.3%) | 2 (5.3%) | 3 (100.0%) |
| **Sub-total** |  | 0 (100.0%) | 19 (100.0%) | 38 (100.0%) | 57 (100.0%) |
| **Total** |  | 118 (31.7%) | 52 (14.0%) | 202 (54.3%) | 372 (100.0%) |

**Table S3. Characteristics and posterior distribution of time to most recent common ancestors estimated for Kenyan HIV-1 clusters.**

| **Cluster name^1^** | **Tips (N)^2^** | **Province** | **Year(s) of diagnosis^3^** | **tMRCA^4^** |
| --- | --- | --- | --- | --- |
| A1.28 | 9 | Nyanza/Nairobi/Coast | 2009-2017 | 1987 |
| A1.18 | 4 | Coast/Nairobi | 2009-2017 | 1993 |
| A1.20 | 4 | Coast/Nairobi | 2006-2016 | 1993 |
| A1.30 | 11 | Coast | 2007-2015 | 1996 |
| A1.32 | 16 | Nyanza/Nairobi/Coast | 2010-2017 | 1996 |
| A1.33 | 19 | Nyanza/Nairobi/Coast | 2015-2017 | 1997 |
| A1.34 | 20 | Nyanza/Nairobi/Coast | 2008-2017 | 1997 |
| A1.31 | 13 | Coast/Nairobi | 2009-2017 | 1998 |
| A1.17 | 4 | Coast | 2006 | 1999 |
| A1.26 | 7 | Coast/Nairobi | 2009-2017 | 1999 |
| A1.29 | 9 | Nyanza/Nairobi | 2016-2017 | 1999 |
| A1.23 | 5 | Coast/Nairobi | 2006-2017 | 2000 |
| A1.16 | 4 | Coast/Nairobi | 2008-2017 | 2001 |
| A1.3 | 2 | Coast | 2010-2013 | 2002 |
| A1.24 | 5 | Coast | 2008-2014 | 2002 |
| A1.25 | 6 | Nairobi | 2016-2017 | 2002 |
| A1.27 | 7 | Nyanza/Nairobi/Coast | 2007-2017 | 2002 |
| A1.15 | 3 | Coast | 2008 | 2003 |
| A1.11 | 2 | Nairobi | 2017 | 2004 |
| A1.5 | 2 | Coast | 2006 | 2005 |
| A1.8 | 2 | Coast | 2006 | 2005 |
| A1.13 | 3 | Nyanza/Nairobi | 2016-2017 | 2005 |
| A1.14 | 3 | Nairobi | 2017 | 2007 |
| A1.19 | 4 | Coast/Nairobi | 2015-2017 | 2007 |
| A1.7 | 2 | Nairobi | 2016-2017 | 2008 |
| A1.9 | 2 | Nairobi | 2016-2017 | 2008 |
| A1.21 | 4 | Coast/Nairobi | 2010-2017 | 2008 |
| A1.10 | 2 | Coast | 2010 | 2009 |
| A1.12 | 2 | Coast | 2014 | 2009 |
| A1.22 | 5 | Coast | 2011-2019 | 2009 |
| A1.1 | 2 | Nyanza | 2016 | 2010 |
| A1.4 | 2 | Nairobi | 2016-2018 | 2010 |
| A1.6 | 2 | Nyanza | 2015-2016 | 2012 |
| A1.2 | 2 | Nyanza | 2015 | 2014 |
| C.1 | 2 | Coast | 2008-2009 | 1988 |
| C.2 | 2 | Nyanza/Coast | 2008-2015 | 1998 |
| C.4 | 5 | Coast/Nairobi | 2010-2017 | 2009 |
| C.3 | 3 | Coast/Nairobi | 2017-2019 | 2014 |
| D.8 | 6 | Nyanza/Coast | 2010-2016 | 1976 |
| D.2 | 2 | Coast/Nairobi | 2016-2017 | 1983 |
| D.7 | 6 | Coast/Nairobi | 2008-2017 | 1988 |
| D.1 | 2 | Coast | 2009 | 2002 |
| D.3 | 2 | Coast/Nairobi | 2013-2017 | 2004 |
| D.5 | 3 | Coast | 2008-2009 | 2007 |
| D.4 | 2 | Coast | 2016-2019 | 2014 |
| D.6 | 5 | Nyanza/Nairobi | 2015-2017 | 2014 |

^1^Clusters are named according to subtype/CRF, and risk group dominating the cluster.

^2^Number of sequences per cluster.

^3^The respective earliest and most recent date (year) of sampling of sequences in the cluster

^4^Estimated tMRCA: Median time to the most recent common ancestor of the cluster.

**Table S4. Phylogeographic inference of HIV-1 migration rates (Bayes factor, BF≥3) between geographic locations in the full Kenyan dataset.**

Bayes factor (BF) support and posterior probability inferred for HIV-1 transmission between geographic locations in the full Kenyan sub-subtype A1, subtype C and subtype D datasets. Only significant transitions (BF≥3) are shown.

|  | **The direction of migration events (from-to)** | **Bayes Factor (BF)** | **Posterior Probability** |
| --- | --- | --- | --- |
| **Migration between provinces** | | | |
| HIV-1 A1 | Coast-to-Nairobi | 3716 | 1 |
|  | Nairobi-to-Nyanza | 3716 | 1 |
|  | Nyanza-to-Coast | 4 | 0.8 |
| HIV-1 C | Coast-to-Nairobi | 268 | 1 |
|  | Coast-to-Nyanza | 8 | 0.9 |
|  | Nyanza-to-Coast | 3 | 0.7 |
|  | Nairobi-to-Coast | 3 | 0.7 |
| HIV-1 D | Nairobi-to-Nyanza | 43 | 1 |
|  | Coast-to-Nairobi | 16 | 0.9 |
|  | Nyanza-to-Coast | 4 | 0.8 |

## **Table S5. Temporal proportion in transitions within and between geographic provinces in the HIV-1 A1 dataset.**

| **Jumps (from – to)^*^** | **Year (Range)** | | | **Total** |
| --- | --- | --- | --- | --- |
|  | **1990-2000** | **2001-2010** | **2011-2020** |  |
| Within-provinces |  |  |  |  |
| Coast-Coast | 22 (91.7%) | 117 (69.2%) | 10 (9.8%) | 149 (50.5%) |
| Nairobi-Nairobi | 0 (0%) | 14 (8.3%) | 56 (54.9%) | 70 (23.7%) |
| Nyanza-Nyanza | 0 (0%) | 4 (2%) | 19 (19%) | 23 (8%) |
| Between provinces |  |  |  |  |
| Coast-Nairobi | 1 (4.2%) | 24 (14.2%) | 5 (4.9%) | 30 (10.2%) |
| Coast-Nyanza | 1 (4.2%) | 6 (3.6%) | 0 (0%) | 7 (2.4%) |
| Nairobi-Nyanza | 0 (0%) | 4 (2.4%) | 11 (10.8%) | 15 (5.1%) |
| Nyanza-Nairobi | 0 (0%) | 0 (0%) | 1 (1%) | 1 (0.3%) |
| Total | 24 (100%) | 169 (100%) | 102 (100%) | 295 (100%) |

^*^Transitions between geographic provinces were summarised from the HIV-A1 trait-annotated maximum clade credibility tree which had denser sampling (number of sequences) and temporal coverage compared to other subtypes.

**FIGURES**

**Figure S1.** **The frequency of HIV-1 subtypes per province and by year (range) of sampling.**

The proportion of HIV-1 subtypes per province distributed into three time periods (i.e. 2006-2010, 2011-2015, and 2016-2019).


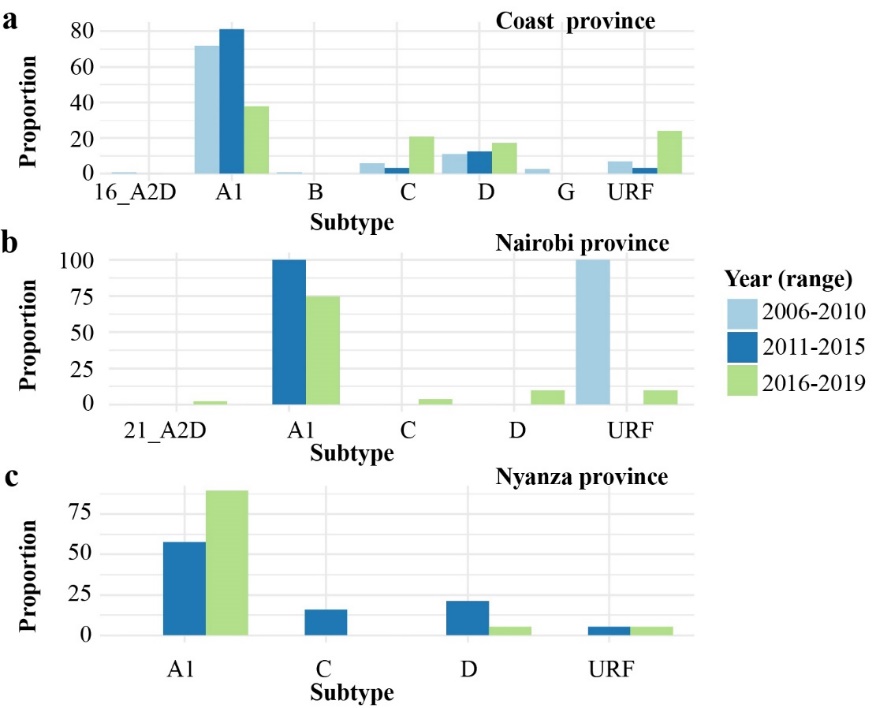


**Figure S2.** **Maximum-likelihood trees used to identify HIV-1 clusters.**

Maximum-likelihood trees used for the identification of MSM HIV-1 clusters. Trees represent A: Sub-subtype A1; B: Subtype C; and C: Subtype D HIV-1 clusters, respectively. Each phylogeny is rooted at the midpoint. Monophyletic clusters with aLRT-SH support ≥0.9 and which have ≥80% sequences from coastal Kenya are highlighted in grey. To enhance cluster visualization, some branches containing either reference sequences or Kenyan sequences that are not part of clusters have been collapsed (shown as black or red triangles, with the recent end of the triangle indicating the latest sampling date. Branch tips within respective clusters are coloured as per geographic province cluster (Orange: Coast; Green: Nairobi; Sky blue: Nyanza; and Black: Reference sequences). Scale bars represent a genetic distance of 0.01 in all phylogenies.

**
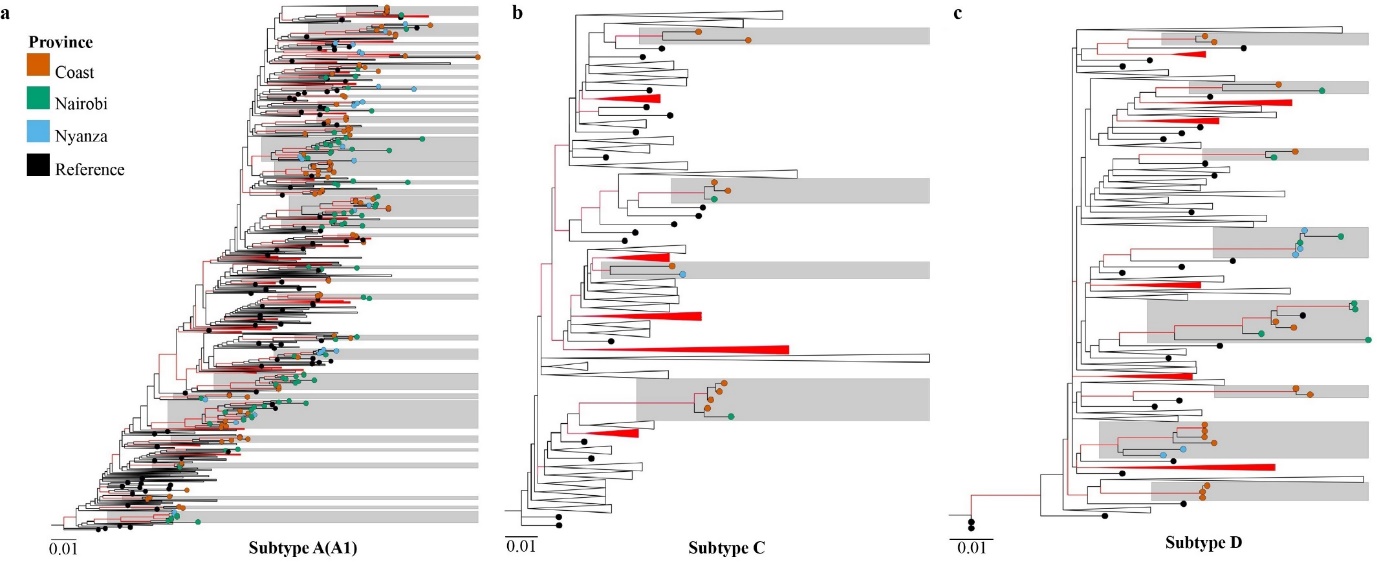
**

**Figure S3. Maximum clade credibility trees used to date clusters.**

Maximum clade credibility (MCC) trees used to determine the time to the most recent common ancestor of the Kenyan HIV-1 clusters. Trees represent A: Sub-subtype A1; B: Subtype C; and C: Subtype D, respectively. To enhance cluster visualization, some branches containing either reference sequences or coastal Kenya sequences that are not part of clusters have been collapsed (shown as non-coloured, black, or red triangles, with the recent end of the triangle indicating the latest sampling date. Branch tips are colour-coded as per geographic province cluster (Orange: Coast; Green: Nairobi; and Sky blue: Nyanza).

**
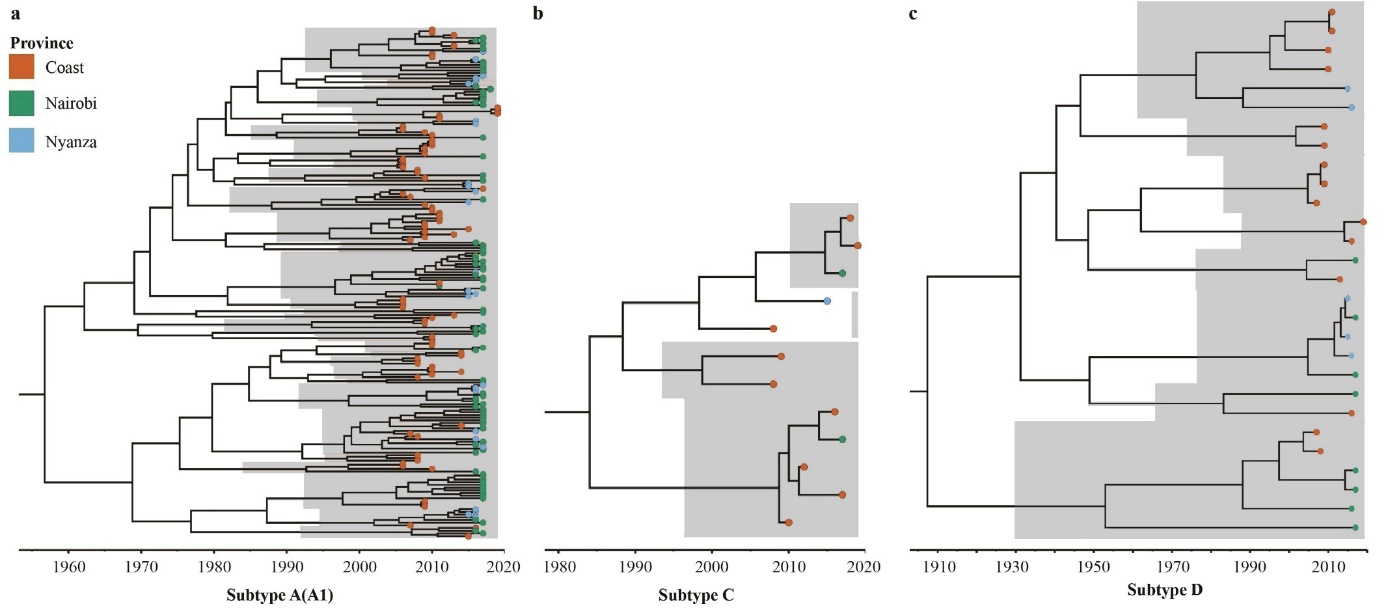
**

**Figure S4.** **The maximum clade credibility tree summary of the Bayesian inference.**

Maximum clade credibility trees revealing phylogeographical estimates of HIV-1 spread in three Kenyan provinces. Trees represent A: Sub-subtype A1; B: Subtype C; and C: Subtype D, respectively. Branch colours correspond to the province of origin as shown in the legend: Orange: Coast; Green: Nairobi; Sky blue: Nyanza.


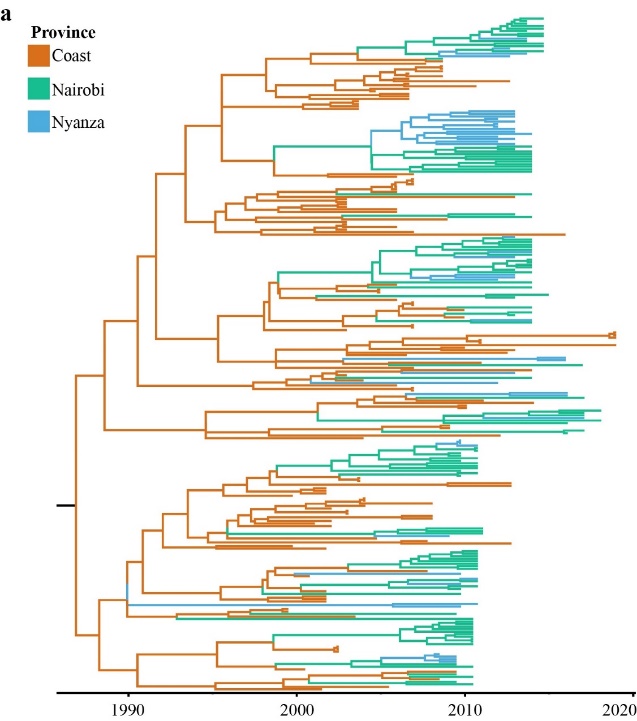


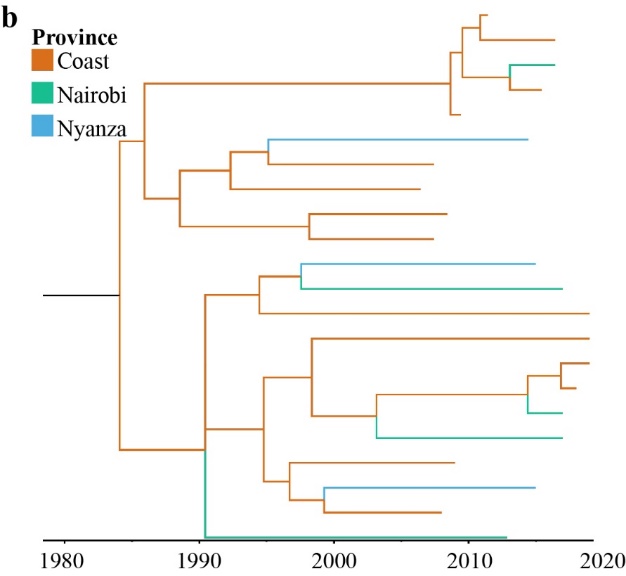


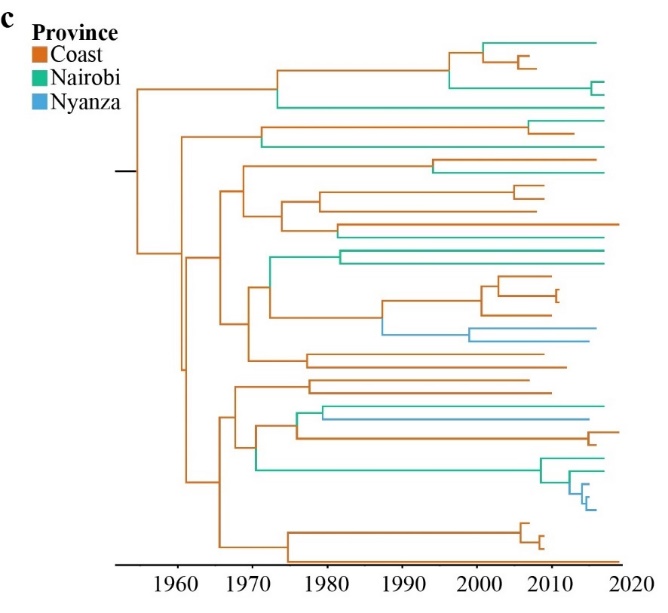


## **Figure S5.** **The estimated proportion and dates of HIV-1 transitions between geographic provinces and risk groups.**

Pirate plots summarising the dates (year) and the frequency of HIV-1 transitions between geographic provinces summarised from trait-annotated maximum clade credibility trees. Plots represent (a) sub-Subtype A1, (b) subtype C, and (c) subtype-D transitions – where group median and interquartile range are coloured by the source of transition (Orange; transitions from Coast, Green; transitions from Nairobi, and Sky-Blue; transitions from Nyanza). Only transitions with a posterior probability higher than 0.90 are plotted. Dots in the pirate plots represent HIV-1 migration events.


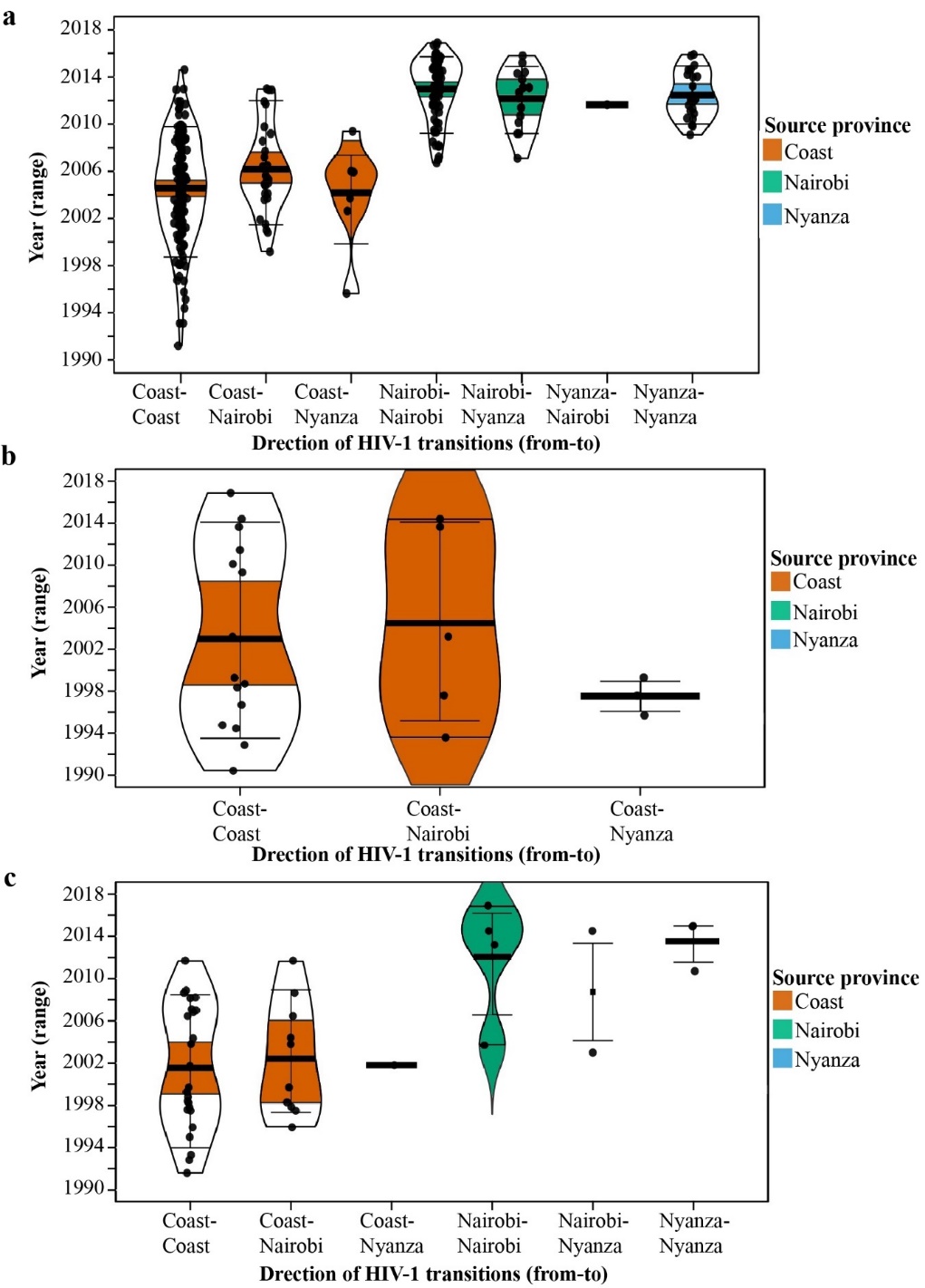

Supplement: Supplementary file 1 [file Data_Sheet_2.docx]
